# Neurobiological mechanism of music improving gait disorder in patients with Parkinson’s disease: a mini review

**DOI:** 10.3389/fneur.2024.1502561

**Published:** 2025-01-03

**Authors:** Ling-Zhi Huang, Zhi Qi

**Affiliations:** ^1^College of Art, Xiamen University, Xiamen, China; ^2^Department of Basic Medical Sciences, School of Medicine, Xiamen University, Xiamen, China

**Keywords:** rhythmic entrainment, central pattern generator, gait, music, Parkinson’s disease

## Abstract

Walking ability is essential for human survival and health. Its basic rhythm is mainly generated by the central pattern generator of the spinal cord. The rhythmic stimulation of music to the auditory center affects the cerebral cortex and other higher nerve centers, and acts on the central pattern generator. By means of rhythm entrainment, the central pattern generator can produce walking rhythm synchronized with music rhythm, control muscle tension, and then regulate human gait. Basal ganglia dysfunction is the main cause of abnormal gait in patients with Parkinson’s disease. Music therapy provides external rhythmic stimulation, recruits neural networks to bypass the basal ganglia and synchronizes gait with external rhythms in both time and space through auditory-motor neural networks, helping to promote the improvement of abnormal gait patterns in patients with Parkinson’s disease.

## 1 Introduction

Walking is one of the most basic movements of human beings. The basic characteristic of walking is the rhythmic movement of alternating left and right foot. Music stimulates the auditory center through its inherent rhythm, and the auditory center interacts with the motor center to activate motor neurons. Activated motor neurons send out signal to contract muscles, coordinating the body and music rhythm to produce synchronous movement (1). Thus, cognitive and motor functions are enhanced and gait is improved (1, 2). Music therapy can improve the gait of patients with traumatic brain injury (3, 4), stroke (5, 6), Parkinson’s disease (PD) (7, 8), and spinal cord injury (9). From a neurophysiological point of view, music therapy provides external rhythmic stimulation, synchronizes gait with external rhythm by promoting internal neural timing, and helps to promote the improvement of gait pattern and improve quality of life.

Gait disorder is a common disabling symptom of PD. The structural and functional connection between auditory area and motor area is the basis of music regulation of walking rhythm. In order to provide new ideas for the application of music-based therapy in the field, various combinations of keywords, including music, music therapy, rhythmic auditory stimulation, gait, Parkinson’s disease, freezing of gait, were used as search terms through Web of Science for the publication period from 1970 until 2024. A total of 26 relevant published papers about effect of music on PD were included in this mini review. This paper focuses on the effect of music on improvement of gait disorder and its neural mechanism in patients with PD.

## 2 The neural mechanism of rhythmical walking and its regulation

### 2.1 Generation of basic rhythm of walking

**Figure 1:**
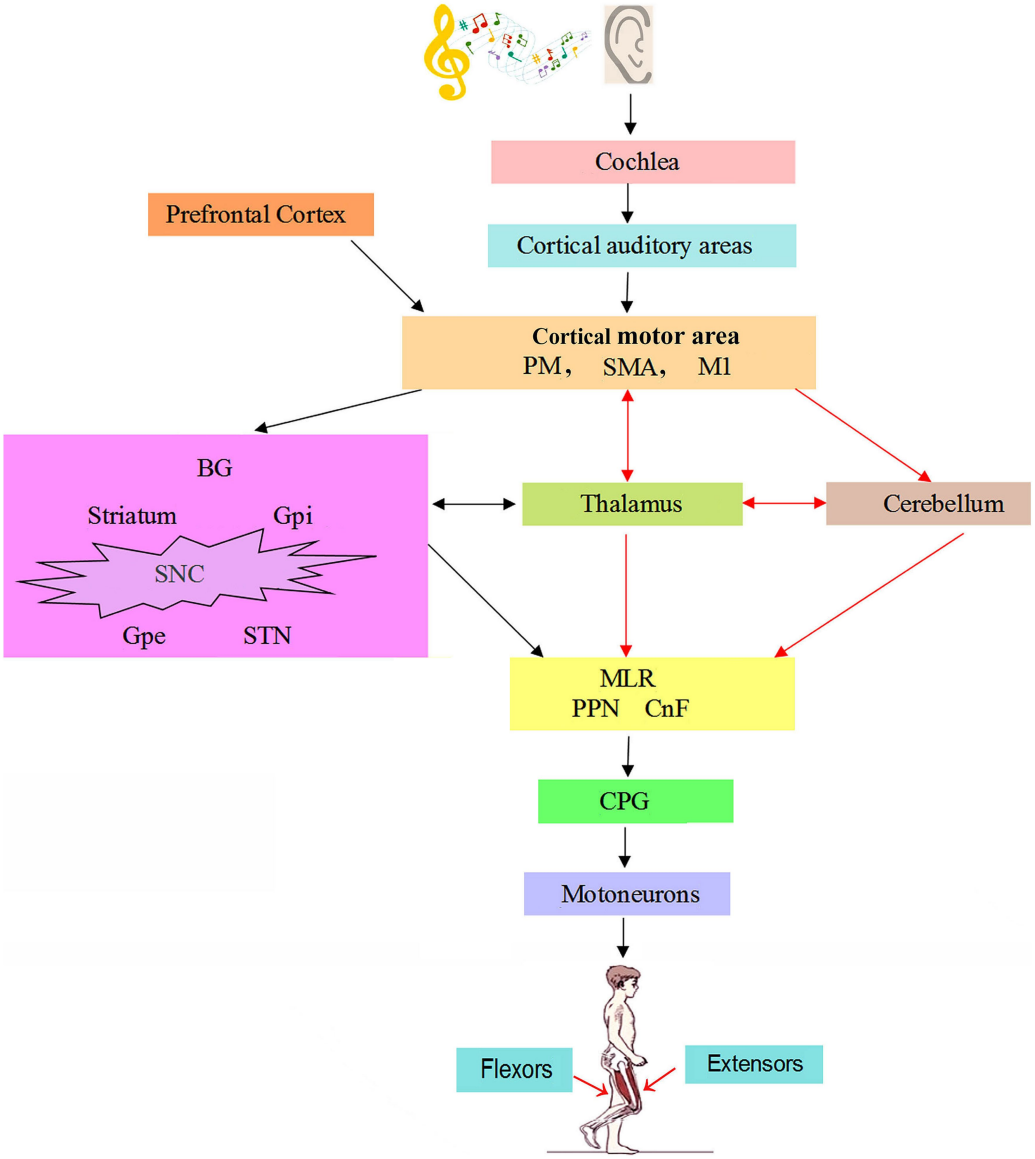
The neural mechanism of music on gait in patients with Parkinson’s disease (22, 25, 38). Auditory information is conveyed from the cochlear nuclei to cortical auditory areas. PM, M1, and SMA generate precise motion and posture control commands. BG receives inputs from the cortical motor area, which is projected to the MLR through different subpopulations of neurons. MLR impinges on reticulospinal neurons to control the spinal CPGs for locomotion. In normal, the brain networks involved in gait regulation include motor cortex, BG, thalamus, cerebellum, MLR and CPG. The striatum in BG receives afferent projections from cortical motor area and thalamus. The cerebellum is conducted downward through their connections with the brainstem and upward to the cortex via the thalamus. In Parkinson’s disease, music guided movements recruits cerebello-thalamo-cortical motor network and thus bypasses the basal ganglia, as shown by the red arrows. CnF = cuneiform nucleus; CPG = central pattern generator; GPe = external globus pallidus; GPi = globus pallidus internus; M1 = primary motor cortex; MLR = mesencephalic locomotor region; PM = premotor area; PMRF = pontomedullary reticular formation; PPN = pedunculopontine nucleus; SMA = supplementary motor area; SNC = substantia nigra pars compacta; STN = subthalamic nucleus.

The initial signal of walking movement comes from the voluntary process of cerebral cortex or the emotional process of limbic system, which requires automatic control of body rhythm movement (10). Neural networks involved in normal gait regulation include the cerebral motor cortex, basal ganglia (BG), thalamus, cerebellum, midbrain motor area, and the brainstem and spinal cord descending system (11). Modification of gait requires the transmission of motor programs from the premotor cortex (PMC) to the brain stem via the reticulospinal system. In response to signals in proprioceptive and skin afferents, the interspinal neuron network modifies locomotor patterns in cooperation with descending signals from the brainstem structures and the cerebral cortex. The flow of information between the BG, cerebellum, and brain stem can automatically regulate muscle tension and skeletal muscle contraction without consciousness, resulting in joint movement and finally rhythmic walking movement (10). The basic rhythm of walking movement is generated by central pattern generators (CPGs) (12–15). The CPG is located in the ventral motor region of the spinal cord and consists of a variety of interneurons located within the spinal cord (16). The generation of rhythm depends on the balance of excitatory and inhibitory neuron activity, and the neural network composed of inhibitory interneurons is the main factor to produce the rhythmical activity of walking. The spinal cord CPG is composed of the spinal cord interneuron network to form a local oscillation network, which generates stable oscillation behavior through the mutual inhibition of neurons to generate stable phase interlock, and controls the rhythmic movement of walking and other related parts of the body through self-excited oscillation. Rhythmic interneuronal activity is sent to secondary interneurons in the intermediate region, which shape the motor pattern of each limb movement (17). Then, the signal is transmitted to the target motor neurons, which orderly activate the extensor and flexor muscles to perform alternating contractions to stimulate the rhythmic movement of the limb (Figure 1).

### 2.2 Neural regulation of rhythmic walking

The rhythmic movement of human walking, after starting, can be repeated spontaneously without the need for continuous control of the brain. However, the seamless and smooth walking movement requires multiple levels such as spinocerebellar, spinocerethalamic, and spinocerethalamic tract to transmit sensory information such as proprioception back to the brain. The CPG receives signals from proprioception and skin and modifies movement patterns to coordinate descending information from the brain stem and cerebral cortex. Functional magnetic resonance imaging (fMRI) of 18 healthy volunteers has shown that the bilateral dorsal premotor cortex is involved in generating temporal prediction of auditory rhythm patterns, which is essential for accurate and precise sensorimotor synchronization (18). In the rhythm processing, the cerebellum is involved in multiple stages from sensory prediction to motor control, while the basal ganglia striatum always plays a role in the preparation of movement (19). A combination of molecular genetics, anatomical tracking, and imaging techniques has successfully identified several spinal cord neurons that make up the CPG neural network, as well as their specific roles during walking and descending input control (20). Electrophysiological and tracking techniques in mice revealed that the brain stem transmits descending commands to the caudal spinal cord, which is responsible for starting, stopping, and regulating the rate of generating rhythmic activity (21). On the one hand, the changes of neural network structure and synaptic strength composed of the CPG are regulated by the control commands of the upper center such as brain, cerebellum and brainstem. On the other hand, the feedback information from vision, vestibular sense and proprioception coordinates the relationship between the CPG and the environment through the neural reflex mechanism, and adjusts the output of the CPG, so that the rhythmic movement pattern has better plasticity and the ability to adapt to real-time changes in the complex external environment.

When walking, somatosensory information is delivered to the central nervous system, where it is processed and integrated in numerous cortical and sub-cortical networks and used to execute motor programs. It is worth noting that the organization of the neural network controlling movement is very similar in different vertebrate species (22), indicating that it is very conducive to vertebrate control of movement in different natural environments. The behavior of vertebrates relies on different neural networks whose activity is controlled by the brain stem command center. The descending inputs from this center initiate, maintain, and stop movement and control speed and direction, which in turn is controlled by BG (23). The BG exerts inhibitory control over several motor areas of the brain stem, which in turn control the CPG that produces basic movement (24). The direct pathway of BG and striatum projection neurons initiates movement, while the indirect pathway inhibits movement through the outer white bulb and subthalamic nucleus. The output of the BG (substantia nigra reticulum and inner white sphere) acts on the midbrain motor area consisting of the peduncular nucleus and the cuneate nucleus, which in turn influences reticulospinal neurons in the lateral accessory giant cell nucleus that controls the CPG of the spinal cord (25). Gait is a complex motor task that involves all levels of the nervous system from cortex to the spinal cord, from locomotor networks to cognitive networks and requires integration of neuromuscular networks (24). Failure of the integration will affect one or more nodes and connections in the locomotor network and cause failure of effective motor output and give rise to gait disorder, such as freezing of gait (FoG) in PD patients (26, 27). The forebrain and in particular the BG are involved in determining motor programs that should be recruited at a given point of time and can both initiate and stop locomotor activity (25). In the resting state, the BG output periodically suppressed the command center of the mesencephalic locomotor region. During walking, it has been suggested that the BG produces precisely timed modulation of low band frequency and beta frequency bands which are disrupted during pathological gait patterns in PD (28). The BG selects and allows the relevant CPGs to enter the motor system in the correct order while inhibiting other behaviors. The BG also controls the turning on and off of spinal cord CPG activity, as well as the magnitude and direction of phase lag. Thus, the BG plays a very important role in determining which CPGs should be active at a given moment (23).

## 3 Neural mechanism of musical rhythm regulating walking movement

### 3.1 Influence of music rhythm on body rhythmic movement

Most people hear music, their body will naturally move, indicating that music rhythm has a strong impact on the body’s rhythmic movement. Even young children are sensitive to the signals carried by the rhythm of music (29). Music is also used in ritual dances to help synchronize movements and communicate between group members (30). Musical rhythm synchronizes the auditory system with the motor system, which can promote the improvement of motor ability (31) and accelerate motor learning (32). Music can enhance the connection between rhythmic auditory perception and motor behaviors (33). In an evolutionary context, music and dance behaviors are closely related to rhythmic synchronized movements and can serve as an effective means of communication and bonding within a group (30). These phenomena suggest that the human ability to perceive rhythm may be selected for coordination among individuals within a group.

A Genome-wide association study can determine genetic variation in a trait, specifically to detect associations between common single nucleotide polymorphisms (SNPS) by testing hundreds of thousands of genetic variants across many genomes (34). To identify the genetic alleles associated with beat synchronization ability in humans, a genome-wide association study of more than 600,000 individuals was recently conducted in humans. The study found that beat synchronization exhibited a high degree of polygenic structure, with 70 “sentinel” SNPS at 69 genomic loci reaching genome-wide significance, and a total of 6,160 SNPS passing the genome-wide significance threshold. These genetic correlations suggest a common genetic structure between beat synchronization and biorhythms such as breathing and movement (35). Music, with its unique rhythm, is one of the ideal ways to drive movement (32). Interestingly, a variety of animals also has the ability to channel body movements into a rhythmic auditory rhythm. Music was present in the common ancestor of humans and chimpanzees about 6 million years ago (36). This conservatism explains the importance of sound rhythm in the regulation of motor rhythm.

### 3.2 The structural and functional connection between auditory area and motor area is the basis of music regulation of walking rhythm

People often follow rhythm when they hear rhythmic music, so there is a deep relationship between the motor center in human brain and the auditory center that perceives rhythmic music. The individual auditory system uses the externalized auricle to capture sound frequency information, and the sound signal is converted into an electrical signal and transmitted from the cochlear auditory nerve to the anterior cochlear ventral nucleus, and finally into the auditory center of the cerebral cortex. The auditory area is adjacent to the motor area, and the two areas are prone to interaction. Results from fMRI have shown that the BG is activated during beat perception of musical rhythms. In people with musical training, there is increased connectivity between the motor and auditory areas of the cerebral cortex (37). Within the cerebral cortex, rhythm and audio-motor interactions occur in a widely distributed and layered network of neurons extending from the brainstem and spinal cord to the cerebellum, BG and cortical ring, thus facilitating the interaction between the auditory and motor systems (38). The extensive connection between auditory and motor systems in the brain promotes the synchronization of rhythmic music and walking movement to produce coordinated movement, which is the neural basis for music to improve movement efficiency (33).

### 3.3 The entrainment of walking movement rhythm by music and its neurobiological mechanism

The discovery of entrainment dates back to 1,666, when Christiaan Huygens noted that two pendulum clocks had independent frequencies or periods of motion when they moved independently. However, the two pendulum clocks interact and eventually synchronize and lock into a stable rhythm or cycle when placed on the same flexible surface (39). Since then, similar phenomena have been observed in various mechanical devices and biological systems, which exhibit rhythmic behavior with periodic oscillations (40). Entrainment is often defined as the process by which two or more biological or mechanical systems interact to synchronize resulting in various forms of temporal coordination (41, 42). Music entrainment is to cause the change of neural oscillation of corresponding frequency in the brain through the perception of the time and hierarchical structure of the music rhythm, and make the movement frequency of human limbs spontaneously synchronize with the external music rhythm.

Studies on sensorimotor synchronization have shown that the sensorimotor cortex, supplementary motor area (SMA) and PMC, as well as the cerebellar thalamic cortex, posterior parietal area and cerebellar network are all involved in the synchronization beat task. CPGs not only produce motor rhythms, but may also promote audio-motor synchronization (43). The highly interactive circuitry of the cerebellum, BG and cerebral cortex supports the transmission of cerebellar time prediction to the thalamo-striator-cortical network (44). Neuroimaging studies have found that motor areas of the brain are activated even when humans passively listen to metric-based rhythms, suggesting that the motor system also plays an important role in processing rhythms (45). During the synchronization of movement and rhythmic music, the frontal parietal network consisting of lateral prefrontal and subparietal regions, BG and cerebellum was more active (46). Music-related activities, such as listening to and making music, promote the connectivity of brain regions involved in a large number of cortical and subcortical structures, thus affecting body motor function (47). Entrainment phenomenon can even occur at the level of neurons. Neuron activity has its own spontaneous oscillation mode, in the absence of external stimulation. However, the spontaneous oscillation mode of the neuron changes, and tends to adapt to the external stimulus when the amplitude, phase, presentation frequency and action time of the external stimulus act on the neuron. When more and more neurons are affected, the corresponding neuron clusters show activity patterns consistent with external stimuli, and can be observed by neuroscientific research methods such as electroencephalography and magnetoencephalography. Entrainment, as a form of communication between organized and connected neurons, is therefore an important principle in neurosciences (48).

## 4 Neurobiological mechanism of music improving gait disorder in PD

### 4.1 Gait disorder symptoms of PD

Gait disorder is a common disabling symptom of PD patients (49). The loss of postural control and altered locomotion patterns in PD cause impaired balance disturbances, falls, tremor, muscular rigidity, bradykinesia, en bloc turns, festination, and FoG, which plays an important role in the quality of life, morbidity, and mortality of patients with PD (50–52). The pathophysiological mechanisms of FoG and postural control involves a large brain network where motor, sensory, and cognitive/emotional systems intersect. Therefore, in 2022, the Journal of the Movement Disorder Society has held a critical panel discussion about gait abnormalities in PD, indicating that it is necessary to develop “systems neuroscience” strategy to study the circuits/networks involved. In most cases, symptoms start in one side of the body with contralateral symptoms appearing within a few years. A quarter to 60% of patients experience freezing of movements usually after several years from onset (53). Therefore, gait recovery is an important goal of rehabilitation programs for patients with PD.

### 4.2 Neural mechanisms of PD

It has been recognized that the loss of dopaminergic signaling in many BG regions is the main cause of the motor symptoms of PD (54, 55). Medium spiny neurons in the direct and indirect pathways of the BG circuit control motor promotion and motor inhibition, respectively, and the two work together to complete normal movement. Dopamine can enhance the activity of the direct pathway through the D1 receptor and inhibit the activity of the indirect pathway through the D2 receptor. In the indirect pathway, external globus pallidus (GPe) GABAergic neurons innervate the same interface nuclei and the neighboring subthalamic nucleus (STN). The activation of indirect pathway inhibits GPe activity, and strengthens basal ganglia GABAergic signal by directly relieving the inhibition of interface nuclei and enhancing excitatory STN input, thereby suppressing movement (55). By conceiving a neurorobotic platform to emulate the key components of walking under well-controlled conditions, it has been shown that the subthalamic nucleus (STN) not only encodes the initiation, termination, and amplitude of leg muscle activation, but also determines the encoding of leg muscle synergies during standing and walking (56). Thus, Parkinson’s disease (PD) is mainly characterized by dopamine depletion in basal ganglia motor circuit, which results in tremor, bradykinesia, reduced movement and slow movement, and difficulty in initiating autonomic movement and other motor disorders of PD (55, 57).

With the progress in recording technology, especially the sensing technology from implanted deep brain stimulation (DBS) electrodes, it is possible to perform simultaneously local field potentials (LFP) recordings and electromyographic recordings of patients during walking through a course that induced FoG episodes. The introduction of DBS not only improved treatment of movement disorders but also led to a better understanding of the pathophysiology of these disorders. These studies indicated that some specific oscillatory patterns relative to the gait cycle are modulated in the cortical–subcortical circuits and locomotor network in PD. For example, subthalamic stimulation modulates both large-scale cortical motor-network activity and synchronization in PD (58). Increases in both low beta and theta subthalamic nucleus activity enhances vulnerability to FoG (59). Recenly, neuromuscular circuit mechanisms of FoG in PD has been revealed by simultaneously recording LFPs of subthalamic nucleus and surface electromyography of antagonistic leg muscles and gait kinematics in patients while walking in straight-line and in freezing (60). This study shows that there are specific activation-deactivation abnormalities of oscillatory activity of the subthalamic nucleus both before and during a freeze and subthalamo-spinal circuits entrain the spinal motor neurons to a defective timing and activation pattern. When PD freezers turning into freezing, reciprocity between antagonistic muscles is disturbed, co-contraction of the antagonist muscles is increased and subthalamo-muscular coherence with the gastrocnemius muscles before the freeze is increased as well.

### 4.3 Neural mechanism of improvement of gait disorder with music in patients with PD

Motor rhythms depend on different brain regions, such as the auditory cortex, inferior parietal and frontal regions. These regions appear to be unaffected by the pathophysiology of PD, but are not easily accessible due to impaired function of the BG, which senses internal timing and internal rhythms. External rhythm cues, on the other hand, include sensory stimuli such as visual and auditory, and can serve as alternative cues for impaired internal time. Cerebellum has the ability to process time perception and movement execution, and cerebellar injury affects the rapid rhythm process. Thus, the rhythmic stimulation of music and the execution of synchronous movements can recruit the cerebellum to recalibrate the motor sensory feedback signals of the internal rhythm to compensate the explicit time disruption for the BG-SMA-PMC (61). The direct projection of cerebellar and other brain regions by auditory cortex provides an anatomical basis for the influence of rhythmic auditory stimulation on cerebellar activities (62).

Rhythm entrainment is one of the important mechanisms for the successful application of rhythmic music stimulation in the rehabilitation of movement disorders (63). Musical rhythm acts as an activation signal to stimulate the motor system of PD patients to synchronize with the musical rhythm. The use of rhythm music stimulation in patients with PD is mainly to activate rhythm entrainment, that is, to lock in the synchronization of auditory rhythm and body movement. In walking exercises synchronized with rhythm music, patients with PD activate motor networks related to rhythm perception, regulate movement through music, and repeat the same action each time, thus accelerating motor learning and increasing the plasticity of learning (64). For example, when patients perform synchronized movements in response to rhythmic musical stimuli, functional neural connections between the auditory cortex and the executive control network and between the executive control network and the cerebellum increase (65). Rhythmic auditory stimulation can effectively improve walking speed and step length, enhance walking stability, decreases the turn time, and reduces the number and the mean duration of the freezing episodes in a FOG eliciting task (66, 67). Music-based movement therapy improved comprehensive motor functions, including gait velocity, the max ankle dorsiflexion in stance, ankle range of motion, and the max extensor moment in stance in PD patients with FOG (68). A systematic review showed that music-based movement therapy can be used to increase gait stability in PD patients with FOG and improve their quality of life (69). A fMRI study performed on the rhythmic motor behavior of PD patients demonstrated that musical stimulation increased functional neural connectivity between the auditory cortex and the executive control network, and between the executive control network and the cerebellum (70). Therefore, the use of rhythmic auditory stimulation to enhance the functional connection between the auditory area and the motor area (71) can promote the synchronization of the walking movement rhythm with the external musical rhythm, improve the gait and continuously improve the motor ability (72) (Figure 1).

## 5 Conclusion

Dysrhythmic walking caused by impaired BG function is one of the main reasons for abnormal gait in PD. The external rhythmic auditory stimulation provided by music can recruits cerebellar and other brain regions. Then, the motor sensory feedback signal of the internal rhythm is recalibrated by rhythm entrainment to compensate for the impaired BG function and induce the walking movement to synchronize with the external musical rhythm. These processes lay the foundation for the treatment and improvement of gait disorders in PD. At present, therapeutic options on PD are not satisfactory. Pharmacological or surgical treatment has a limited effect on alleviating PD symptoms (51). Therefore, a variety of rehabilitation strategies has been used to improve symptoms in PD patients. The combination of conventional treatment and non-pharmacological intervention may be a new direction for the rehabilitation of PD patients and will have a significant beneficial impact on the quality of life of PD patients (73, 74). Studying the neuronal activity in the relevant brain area by using DBS technique to record LFP of neurons during music stimulation will be helpful to illustrate the neurobiological mechanism of music stimulation. On the other hand, musical stimulation is a low cost, easy to control, simple and feasible neuroregulation method, while rhythm is an inherent characteristic of music that can cause synchronization of neural network behaviors. Therefore, combining music therapy with other therapies, such as DBS, is a topic worthy of future research.
